# Prognostic and clinicopathological value of poly (adenosine diphosphate-ribose) polymerase expression in breast cancer: A meta-analysis

**DOI:** 10.1371/journal.pone.0172413

**Published:** 2017-02-17

**Authors:** Weiqiang Qiao, Linlin Pan, Changgui Kou, Ke Li, Ming Yang

**Affiliations:** 1 Department of Breast Surgery, First Hospital of Jilin University, Changchun, China; 2 Department of Epidemiology and Biostatistics, School of Public Health, Jilin University, Changchun, China; 3 Department of Emergency, First Hospital of Jilin University, Changchun, China; Azienda Ospedaliera di Cremona, ITALY

## Abstract

**Background:**

Previous studies have shown that the poly (adenosine diphosphate-ribose) polymerase (PARP) level is a promising indicator of breast cancer. However, its prognostic value remains controversial. The present meta-analysis evaluated the prognostic value of PARP expression in breast cancer.

**Materials and methods:**

Eligible studies were retrieved from the PubMed, Web of Science, Embase, and Cochrane Library databases through July 20, 2016. Studies investigating PARP expression as well as reporting survival data in breast cancer were included. Two independent reviewers carried out all literature searches. The pooled relative risk (RR) and hazard ratio (HR) with 95% confidence interval (95% CI) were applied to assess the association between PARP expression and the clinicopathological features and survival outcome in breast cancer.

**Results:**

A total of 3506 patients from eight eligible studies were included. We found that higher PARP expression indicated a worse clinical outcome in early stage breast cancer, with a HR of 3.08 (95% CI, 1.14–8.29, P = 0.03) for disease-free survival and a HR of 1.82 (95% CI, 1.20–2.76; P = 0.005) for overall survival. Moreover, increased PARP expression was significantly associated with higher nuclear grade (RR, 1.51; 95% CI, 1.12–2.04; P = 0.008) in breast cancer. A similar correlation was detected in triple-negative breast cancer (TNBC; RR, 1.81; 95% CI, 1.04–3.17; P = 0.04).

**Conclusions:**

Our findings indicated that elevated PARP expression correlated with worse prognosis in early stage breast cancer. Furthermore, high PARP expression was associated with higher nuclear grade and TNBC.

## Introduction

Breast cancer is the most common cancer occurring among women worldwide, and 1.7 million new cases and 521,900 deaths were reported in 2012 according to global cancer statistics. Moreover, this malignancy has become the leading cause of cancer-related death among females in developing countries[1]. Breast cancer significantly impacts female health in many ways; therefore, evidence-based clinical practices aimed at decreasing the morbidity and mortality in the real setting are critical. Currently, precise breast cancer treatment according to molecular subtypes, which are determined by estrogen receptor (ER), progesterone receptor (PR), and human epidermal growth factor receptor 2 (HER2) expression and Ki67 status, is highly recommended by the oncological community[2]. However, in special cases such as triple-negative breast cancer (TNBC), which is defined by a tumor that lacks expression of ER, PR, and HER2, therapeutic targets are lacking, and the cancer shows aggressive clinical behavior[3]. Novel biomarkers for accurate diagnosis, treatment, and prognosis as well as effective molecular targets have yet to be found for this subtype of breast cancer.

The poly (ADP-ribose) polymerases (PARPs) are a family of enzymes constituting 18 proteins and play significant roles in DNA damage responses and maintaining the stability of the genome[4, 5]. Recently, researchers reported that PARP inhibitors could be used to treat TNBC[6]; however, the relationship between the PARP expression level and TNBC is unclear. Recent studies have suggested that PARP over-expression is a poor prognostic marker for breast cancer survival[7, 8]; nonetheless, contradictory evidence also has been reported[9]. These conflicting conclusions regarding the association between PARP expression and breast cancer clearly indicate the need for further investigation before application of this marker in future breast cancer diagnosis, treatment, and prognosis.

In order to clarify these inconsistent results, we conducted a meta-analysis to quantitatively evaluate the relationship between PARP expression and the prognosis of breast cancer. Additionally, we comprehensively assessed the association of PARP expression with clinicopathological characteristics of breast cancer. In particular, the correlation of PARP expression and pathological complete response (pCR) after neoadjuvant chemotherapy in breast cancer was systematically reviewed.

## Materials and methods

The present study was conducted in accordance with the Preferred Reporting Items for Systematic Reviews and Meta-Analyses (PRISMA) guideline (S1 File).

### Search strategy

Eligible studies were identified by searching the literature in the PubMed, Web of Science, Embase, and Cochrane Library databases through July 20, 2016. Manual searches of the reference lists for the American Society of Clinical Oncology Breast Cancer Symposiums and San Antonio Breast Cancer Symposiums of the past 5 years also were conducted. The search strategies combined MeSH terms with free-text words to increase comprehensiveness. The primary terms used in the literature search included “PARP”, “breast neoplasms”/“breast cancer”/“breast carcinoma”, and “survival”/“outcome”/“pCR”/“pathological complete response”. We attempted to contact the authors to acquire further information, if the published data were insufficient for analysis.

### Inclusion and exclusion criteria

The included articles satisfied the following criteria: (i) all enrolled breast cancer patients were diagnosed by pathological examination; (ii) the correlation among PARP expression, clinicopathological parameters, and survival was discussed; and (iii) sufficient data were presented to calculate the relative risk (RR) and hazard ratio (HR) with 95% confidence interval (95% CI) for clinicopathological features and the survival rate. The exclusion criteria were as follows: (1) duplicate publication; (2) non-human research and non-English publications; (3) conference publications; or (4) reviews, books, case reports, or letters. When several published articles with data obtained from the same trial were identified, only the most recent or most informative one was selected.

### Data extraction

Two independent reviewers (Weiqiang Qiao and Linlin Pan) conducted all literature searches. Cases of inconsistency and disagreements were resolved through discussion and consensus of all reviewers. Data including authors, time of publication, country, breast cancer subtype, patient number, median age, median follow-up, detection strategy, PARP phenotype, cut-off values for PARP expression, definition of pCR, chemotherapy regimen, clinical outcome, as well as analyzable data provided in the tables and figures were systematically extracted. When survival data were presented only in a Kaplan-Meier curve, effort was made to contact the authors for further information, and if no response was provided for precise evaluation, the survival data were analyzed with Engauge Digitizer, version 8 (accessible at http://markummitchell.github.io/engauge-digitizer/). The quality of the included studies was evaluated based on the Newcastle Ottawa Scale (NOS)[10]. A study was judged according to eight items that were categorized into three perspectives: the selection of enrolled groups, the comparability of the groups and the ascertainment of exposure/outcome, which resulted in a score scale from 0 to 9. Any study with 7 or more stars was considered as high quality research.

### Statistical analysis

Survival analysis was performed based on the strategy introduced by Tierney et al.[11]. All analyses and figures were obtained using Review Manager software, version 5.3 (Cochrane Collaboration, Copenhagen, Denmark) and STATA, version 12.0 (Stata Corporation, College Station, TX, USA). Funnel plot asymmetry was not used to investigate publication bias, since less than 10 studies were included. Begg’s rank correlation was applied instead[12], in which a P value of less than 0.05 was considered to indicate significant potential publication bias. A HR greater than 1 represented an adverse survival benefit for patients with PARP over-expression, while a HR of less than 1 indicated better survival. The association between PARP level and the clinicopathological characteristics of breast cancer, including pCR, age, tumor size, nuclear grade, lymph node status, TNBC, and the expression of ER, PR, HER2, Ki67, and BRCA1 were assessed using RRs and the corresponding 95% CIs. A P value of ≤ 0.1 in the Cochran Q test and an I^2^ statistic >50% were considered to be of significant heterogeneity[13]. The fixed effects model was applied in the initial analysis, and if notable heterogeneity existed, the random effects model was employed[14]. We performed sensitivity analysis by omitting each single study to investigate the robustness of the results. Meta-regression analyses was conducted to explore the sources of heterogeneity. All statistical tests were two-sided, and a P value <0.05 was considered statistically significant.

## Results

### Search results

**Fig 1:**
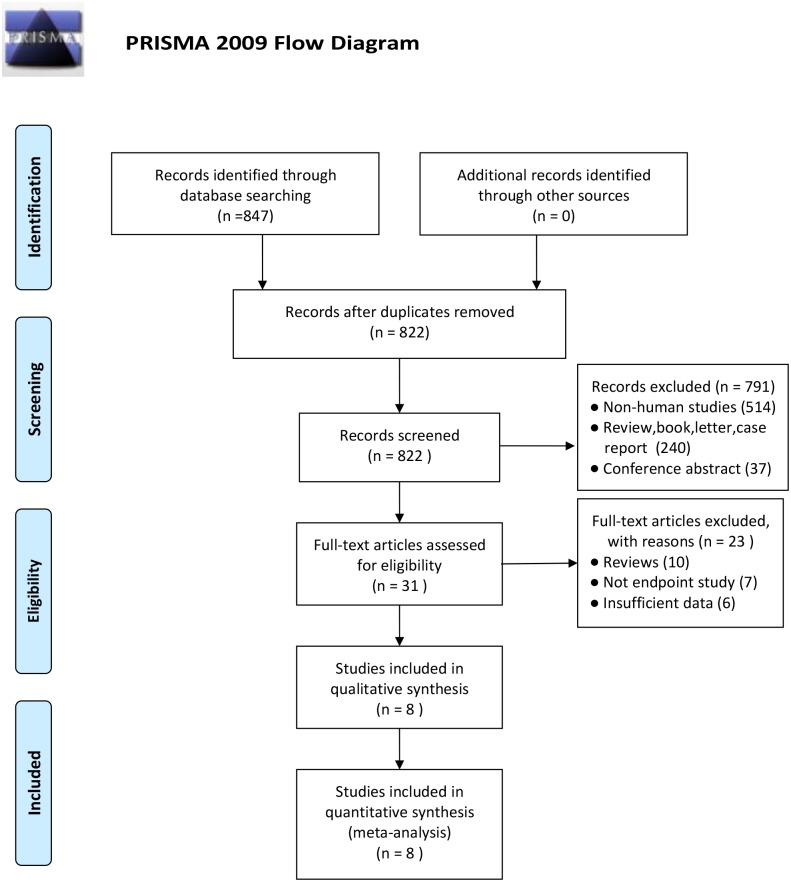
Flow diagram for identification of retrieved publications. *From*: Moher D, Liberati A, Tetzlaff j, Altman DG, The PRISMA Group (2009). *Preferred* Reporting items for Systematic Reviews and *Meta*-*Analyses*: The PRISMA Statement. PLOS Med 6(7):e1000097. doi: 10.1371/journal.pmed1000097 For more information, visit www.prisma-statement.org.

The initial search identified 847 publications using our search strategy (S2 File). Among them, 25 duplicates were excluded. Subsequent screening of the titles and abstracts excluded another 791 researches, as they did not fulfill our inclusion criteria, leaving 31 articles for full review. After reviewing the complete text of the remaining 31 articles, 23 were excluded for the following reasons: review (10), no study endpoint (7), and insufficient data (6). Finally, a total of 3506 patients from eight eligible studies were included[7–9, 15–19]. The details of the selection process are presented in Fig 1. A study by Aiad et al.[9] analyzed the association between nPARP-1, cPARP-1 expression, and survival outcome in breast cancer. Therefore, the eight eligible publications actually presented nine studies.

### Characteristics of eligible studies

The basic characteristics of the enrolled studies are presented in Table 1. All studies were published between 2011 and 2016, and the number of participants in each ranged from 83 to 1269. The PARP expression level was investigated in tumor tissues in all nine studies, with PARP expression detected by immunochemical (IHC) staining in eight studies and by oligonucleotide microarrays in the others. The PARP phenotypes were a variety of PARP-1, nPARP-1, and cPARP. Each study used a different cut-off point, which subsequently impacted the rates of positive PARP expression. The median follow-up ranged from 4 to 8.3 years. All studies showed a significant association between the expression of PARP and clinicopathological parameters and prognosis of breast cancer. The eligible observational studies included survival data for cohort studies, as for quality assessment, all the involved studies were found to be of high quality (S1 Table).

**Table 1:** Baseline characteristics of the included publications.

Publication	Year	Country	Cancer subtype	No. of patients	Median age (years)	Median follow-up (years)	Detection method	PARP phenotype	Cut-off value (low/high expression)	Definition of pCR	Chemotherapy regimen	Outcome
Aiad(1)	2015	Egypt	LABC(cT4, cN0-N2,cM0)	84	53	NR	IHC	nPARP-1	X-tile software,low (<10%),high (≥10%)	MP grades 4/5	FEC	OS/pCR
Aiad(2)	2015	Egypt	LABC(cT4, cN0-N2,cM1)	84	53	NR	IHC	cPARP-1	X-tile software,low (<70%),high (≥70%)	MP grades 4/5	FEC	OS/pCR
Donizy	2014	Poland	stage II ductal BC	83	55.2	NR	IHC	PARP-1	IRS,low (<6),high (≥6)	NR	NR	OS
Goncalves	2011	France	invasive adenocarcinoma BC	1115	NR	8.3	oligonucleotide microarrays	PARP-1	ratio of expression tumor/NB,low (<2),high (≥2)	NR	CMF/AC/AC-T	OS
Green	2015	UK	stage I–III operable BC	1269	NA	NR	IHC	PARP-1nc	H-score,low (≤10),high (>10)	NR	NR	DFS
Mazzotta	2016	Italy	primary operable invasive BC	114	53	4.7	IHC	PARP-1	QS,low (0–9),high (10–18)	NR	NR	DFS/OS
Minckwitz	2011	German	invasive adenocarcinoma BC	313	NR	4.8	IHC	cPARP	IRS,negative (0–2),high (10–18)	ypT0/is ypN0	anthracycline/taxane-based	DFS/OS/pCR
Rojo	2012	Spain	operable BC	330	58	8.5	IHC signal intensity scanning method	PARP-1	optical density of ≥39 970	NR	CMF/hormonal therapy	DFS/OS
Zhai	2015	China	invasive BC	198	53	4	IHC	nPARP-1	QS,low (0–9),high (10–18)	ypT0/is	anthracycline/taxane based	OS/pCR

LABC, locally advanced breast cancer; BC, breast cancer; NR, not reported; IHC, immunohistochemistry; PARP, poly (adenosine diphosphate-ribose) polymerase; pCR, pathological complete response; DFS, disease-free survival; OS, overall survival

### Outcomes

**Fig 2:**
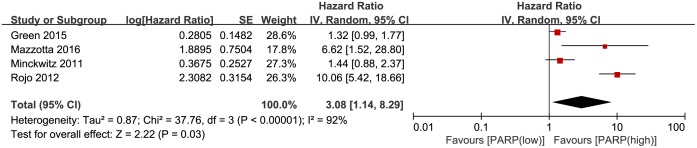
Forest plot for studies investigating the hazard ratio (HR) for PARP expression and disease-free survival (DFS) in breast cancer.

**Fig 3:**
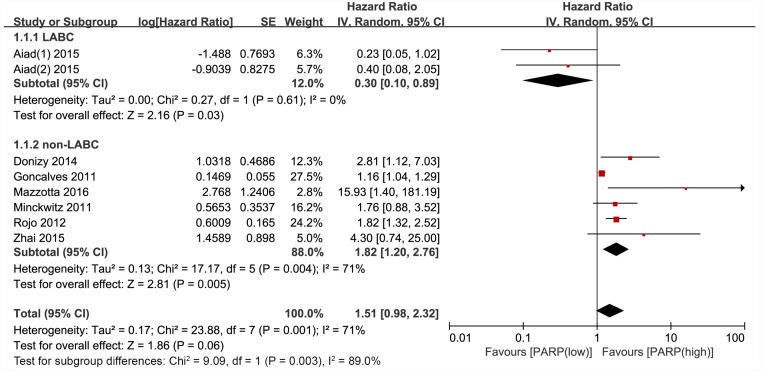
Forest plot for publications assessing the hazard ratio (HR) for PARP expression and breast cancer overall survival (OS).

Four studies reported disease-free survival (DFS). The DFS was notably worse in the group with higher PARP expression compared with that in the lower expression group (HR, 3.08; 95% CI, 1.14–8.29; P = 0.03; Fig 2). The random effects model was applied because of significant heterogeneity (P<0.00001, I^2^ = 92%). Eight articles reported overall survival (OS), and no significant relationship was revealed between PARP expression and OS in breast cancer (HR, 1.51; 95% CI, 0.98–2.32; P = 0.06) (Fig 3). The random effects model was also applied because the heterogeneity was evident among these studies (P = 0.001, I^2^ = 71%). However, the PARP expression in the subgroup of patients with non-locally advanced breast cancer (non-LABC) was significantly related to OS (HR, 1.82; 95% CI, 1.20–2.76; P = 0.005).

**Fig 4:**
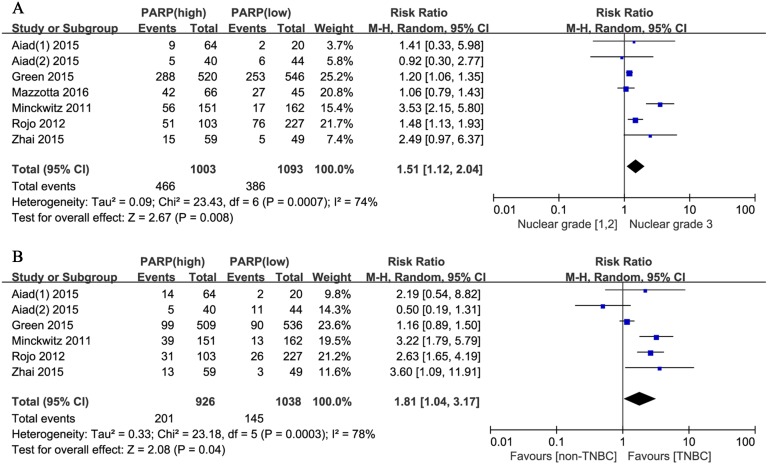
Forest plots for enrolled studies showing positive associations between the PARP expression level and (A) nuclear grade (3 vs. 1 and 2) and (B) triple-negative breast cancer (TNBC) (positive vs. negative).

**Fig 5:**
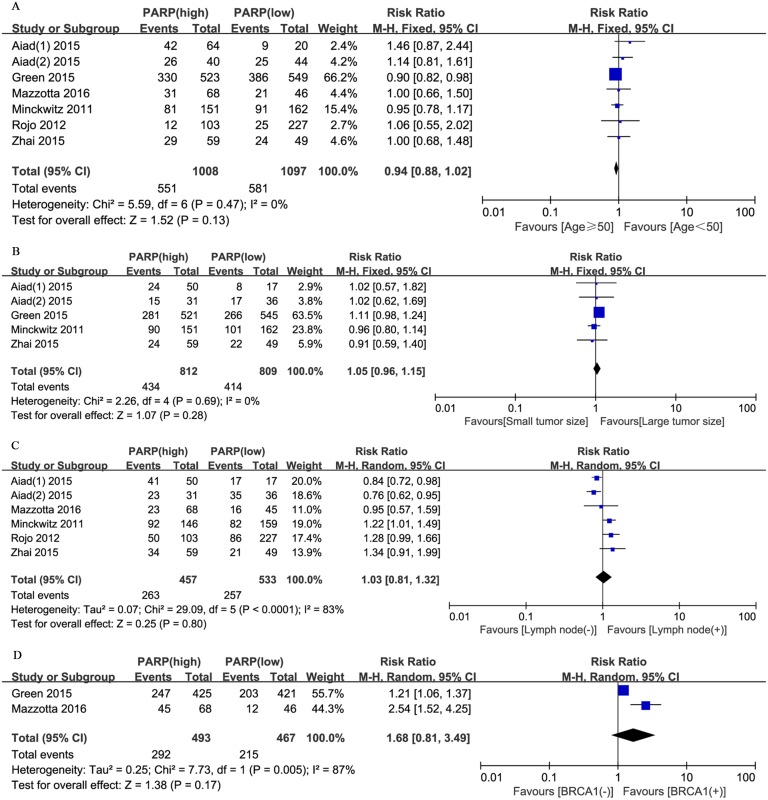
Forest plots for enrolled studies showing that there were no associations between PARP expression and (A) age (≥50 vs. <50 years), (B) tumor size (large vs. small), (C) lymph node metastasis (positive vs. negative), and (D) BRCA1 status (positive vs. negative).

**Fig 6:**
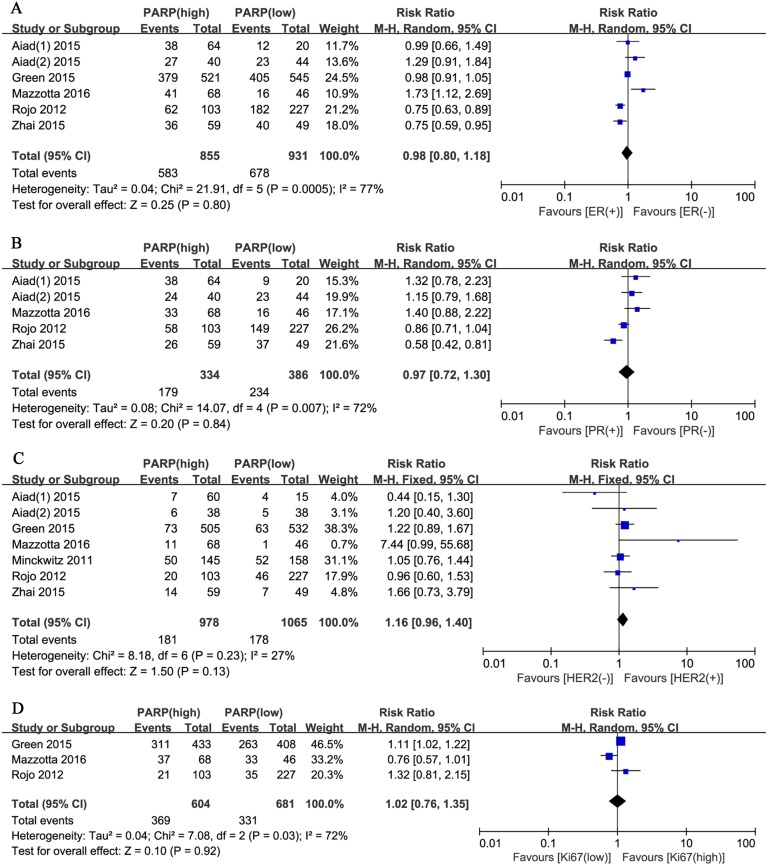
Forest plots for enrolled studies showing that there were no associations between PARP expression and (A) ER expression (positive vs. negative), (B) PR expression (positive vs. negative), (C) HER2 expression (positive vs. negative), and (D) Ki67 level (high vs. low).

**Fig 7:**
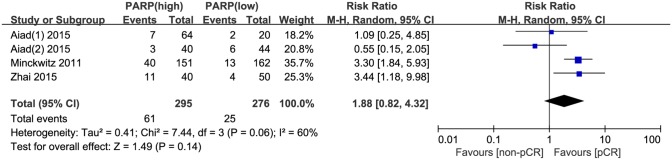
Forest plot for enrolled publications showing that there was no correlation between PARP level and pCR in breast cancer.

Moreover, we evaluated the relationship between positive PARP expression and the clinicopathological characteristics of breast cancer. The nuclear grade of breast cancer was reported in seven studies. The pooled RR was 1.51 (95% CI, 1.12–2.04; P = 0.008; Fig 4A). The random effects model was applied because of significant heterogeneity (P = 0.0007, I^2^ = 74%). In addition, the pooled results from six studies reporting on TNBC showed a pooled RR of 1.81 (95% CI, 1.04–3.17; P = 0.04; Fig 4B). The random effects model was also applied because of obvious evidence of heterogeneity (P = 0.0003, I^2^ = 78%). However, no significant correlation was revealed between the PARP expression level and other clinicopathological parameters, including age (RR, 0.94; 95% CI, 0.88–1.02; P = 0.13; Fig 5A), tumor size (RR, 1.05; 95% CI, 0.96–1.15; P = 0.28; Fig 5B), lymph node metastasis (RR, 1.03; 95% CI, 0.81–1.32; P = 0.80; Fig 5C), BRCA1 status (RR, 1.68; 95% CI, 0.81–3.49; P = 0.17; Fig 5D), ER status (RR, 0.98; 95% CI, 0.80–1.18; P = 0.80; Fig 6A), PR status (RR, 0.97; 95% CI, 0.72–1.30; P = 0.84; Fig 6B), HER2 status (RR, 1.16; 95% CI, 0.96–1.40; P = 0.13; Fig 6C), Ki67 status (RR, 1.02; 95% CI, 0.76–1.35; P = 0.92; Fig 6D), and pCR (RR, 1.88; 95% CI, 0.82–4.32; P = 0.14; Fig 7).

### Sensitivity analysis

Sensitivity analysis was performed by deleting one study at a time. The results indicated that the corresponding pooled HRs of DFS and OS did not significantly change, which suggests the robustness of the results (S1 Fig). However, one study (Green, 2015) influenced the robustness of the results for age and tumor size (S2C and S2D Fig). Results were robust and reasonable for other clinicopathological characteristics, including nuclear grade, TNBC, lymph node, BRCA1, ER status, PR status, HER2 status, Ki67 status and pCR (S2–S4 Figs).

### Meta-regression

Meta-regression was conducted on survival outcome. None of covariates identified statistically significant effect on DFS (S2 Table), but cut-off of PARP level was a potential source of heterogeneity for OS (P = 0.04) (S3 Table).

### Publication bias

No evidence of publication bias was detected with respect to DFS and OS using Begg’s rank correlation (DFS, p = 0.308; OS, p = 1.000).

## Discussion

To our knowledge, this is the first comprehensive systematic review and meta-analysis assessing the correlation of PARP expression with breast cancer prognosis based on published studies, even though a meta-analysis of the role of PARP mRNA expression in human breast cancer was performed in 2011[15]. We found that the pooled HR for DFS according to PARP expression in breast cancer patients was 3.08 (95% CI, 1.14–8.29, P = 0.03), which demonstrated that patients with higher PARP expression experienced significantly worse DFS compared with that in the lower expression group. Moreover, the PARP expression level did not contribute to improved OS (HR, 1.51; 95% CI, 0.98–2.32; P = 0.06). However, such a difference was detected in the non-LABC subgroup (HR, 1.82; 95% CI, 1.20–2.76; P = 0.005). In the stratification analysis, we found that the majority of patients included in the subcategory had early stage breast cancer, and those who presented higher PARP expression showed significantly poorer DFS and OS. In general, PARP over-expression was associated with poor survival in early stage breast cancer patients, while a similar result was not observed in the advanced breast cancer subgroup.

As indicated above, PARP over-expression was correlated with worse prognosis in early stage breast cancer; this phenomenon justified an investigation of the feasibility of using PARP inhibitors to improve survival in breast cancer patients. However, predictive markers for PARP inhibitors against breast cancer are unclear. One study detected PARP1 over-expression after PARP1 inhibitor treatment, but the corresponding survival correlation was not analyzed[20]. A meta-analysis indicated that PARP inhibitors in cancer therapy provided better PFS with few toxicities, particularly in patients with BRCA absence. However, OS was not affected after similar treatment in the same group of patients. In addition, PARP inhibitors failed to to improve the PFS and OS in a breast cancer subgroup[21]. A phase II clinical study found that dual therapy using the PARP inhibitor iniparib and gemcitabine plus carboplatin prolonged the median PFS from 3.6 months to 5.9 months and the median OS from 7.7 months to 12.3 months in patients with metastatic TNBC[22]. However, a subsequent phase III study failed to obtain similar positive results[23]. A possible reason was that the dual therapy might only contribute to the DNA-damaging group rather than the whole mTNBC population.

We also investigated the relationship between PARP expression and clinicopathological features in breast carcinoma. The pooled RRs were significantly correlated with higher nuclear grade and TNBC, while no significant association was revealed between PARP expression and other clinicopathological factors, including age, tumor size, lymph node metastasis, BRCA1 status, ER status, PR status, HER2 status, and Ki67 status. Several studies proved that advanced nuclear grade was a poor prognosis factor in breast cancer[7, 18]; likewise, some researchers found poorer survival outcome in TNBC compared with other breast tumor subtypes[3, 24, 25]. Therefore, we suggest that some of the patients with a higher nuclear grade or TNBC may benefit from PARP inhibitor treatment, and PARP expression could be a predictive marker for PARP inhibitor therapy. However, such a hypothesis has not yet been approved with the currently available data. Intriguingly, other markers including HP1β and Ets-1 expression showed predictive potential for PARP inhibitor therapy[26,27].

Our study further showed no association between PARP expression and pCR in breast cancer. Previous studies reported that the pCR rate could be an indicator of prognosis in breast cancer[28–30]; while contradictory results did not validate pCR as a surrogate endpoint for improved survival in breast carcinoma[31, 32]. Nevertheless, consistent findings showed that neoadjuvant chemotherapy (NAC) could significantly downstage the primary tumor and improve the rate of successful breast-conserving surgery[33]. A phase II study reported a pCR rate of 33.3% among TNBC and BRCA1/2 mutation patients treated with neoadjuvant therapy with a combination of gemcitabine, carboplatin, and a PARP1 inhibitor[34]. However, further clinical trials detecting the association between PARP inhibitors and PARP protein expression are necessary before these targets are applied in real clinical settings.

We admit that there were several limitations in this meta-analysis. First, some survival data were only provided by the Kaplan–Meier curve, and in such cases, HRs were extracted by Engauge Digitizer software; nonetheless, the approximation might not provide accuracy and robust estimation of the results. Secondly, the breast cancer stage, detection method, PARP phenotype, cut-off level, and treatment regimen varied in enrolled studies. Collectively, these factors created significant heterogeneity. Finally, the number of studies and cases included in this article were relatively small, which could affect the power of statistical analysis.

In conclusion, our findings demonstrate that the PARP expression level was associated with poor prognosis and survival outcome in early stage breast cancer. In particular, a high PARP expression level was associated with a higher nuclear grade and TNBC. Therefore, we suggested that some patients with higher PARP expression level may benefit from PARP inhibitor therapy. However, this inference has not yet been proved, and future randomized controlled trials with large sample sizes are required to clarify the prognostic value and efficacy of PARP expression in predicting PARP inhibitor therapy outcome in breast cancer.

## Supporting information

S1 Fig Sensitivity analysis of (A) DFS and (B) OS. (TIF)

S2 Fig Sensitivity analysis of (A) nuclear grade, (B) TNBC, (C) age and (D) tumor size. (TIF)

S3 Fig Sensitivity analysis of (A) lymph node, (B) BRCA1, (C) ER status and (D) PR status. (TIF)

S4 Fig Sensitivity analysis of (A) HER2 status, (B) Ki67 status and (D) pCR. (TIF)

S1 File Completed 2009 PRISMA checklist. (DOC)

S2 File Search strategy. (DOC)

S1 Table Results of quality assessment. (DOC)

S2 Table Results of meta-regression analysis exploring the source of heterogeneity with DFS. (DOC)

S3 Table Results of meta-regression analysis exploring the source of heterogeneity with OS. (DOC)
