# Supplementary figures and images for: Prognostic and clinicopathological value of poly (adenosine diphosphate-ribose) polymerase expression in breast cancer: A meta-analysis
Source: PLoS One. 2017 Feb 17;12(2):e0172413. doi: 10.1371/journal.pone.0172413 (PMC5315304; doi:10.1371/journal.pone.0172413)

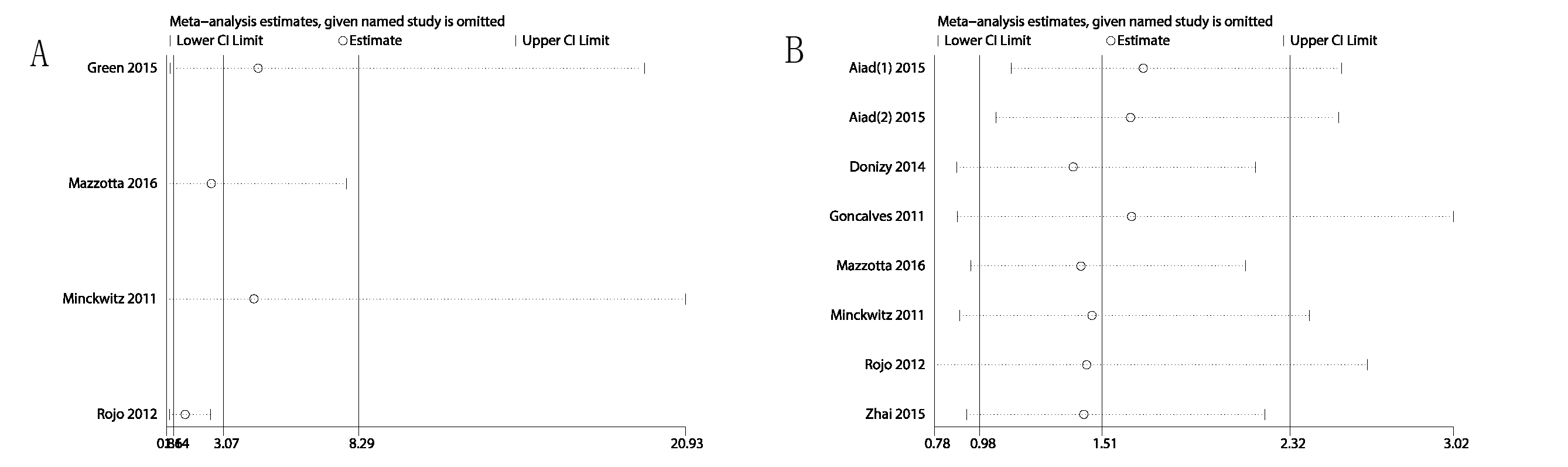

Supplement: S1 Fig — (TIF) [file pone.0172413.s001.tif]

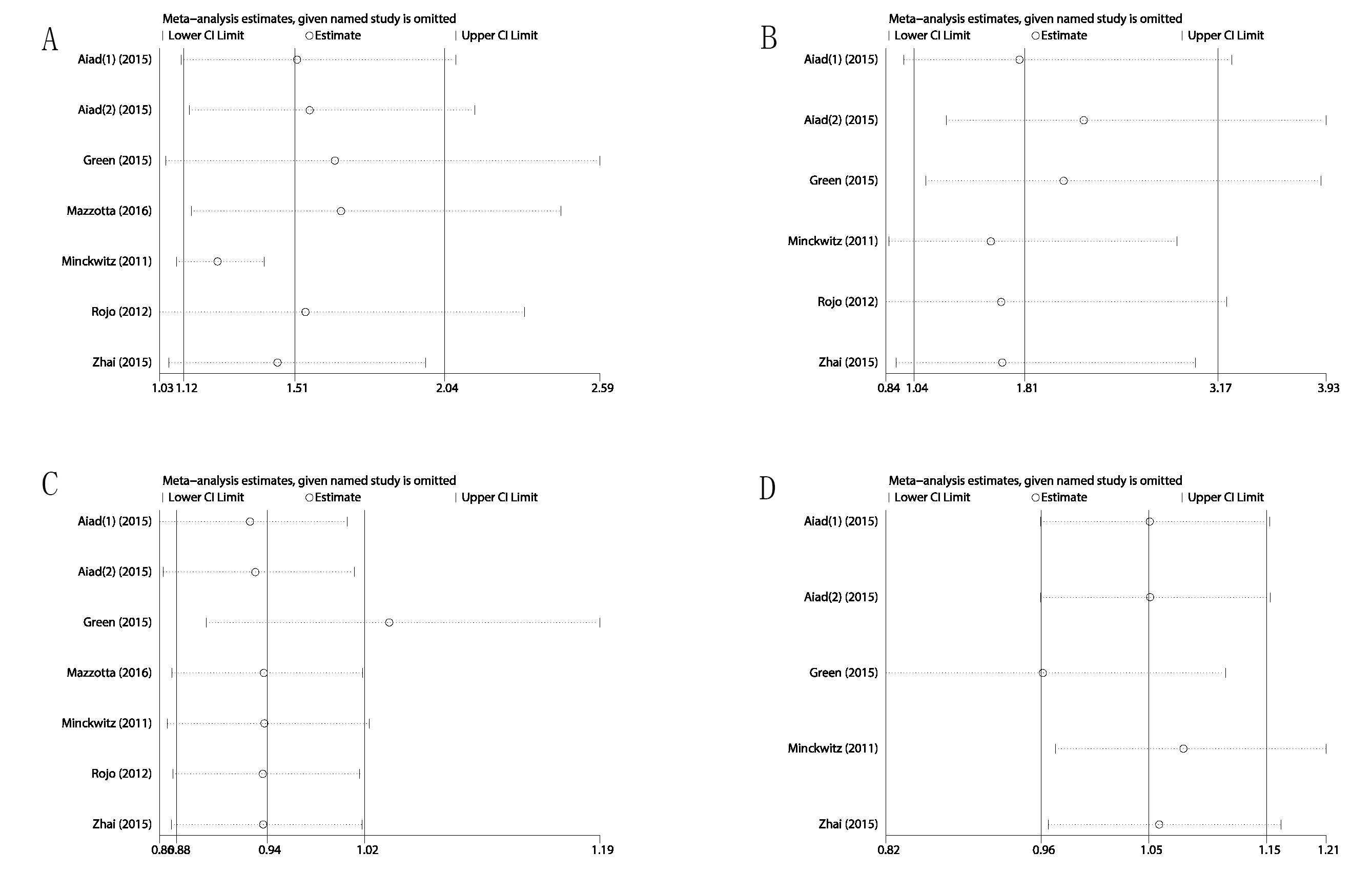

Supplement: S2 Fig — (TIF) [file pone.0172413.s002.tif]

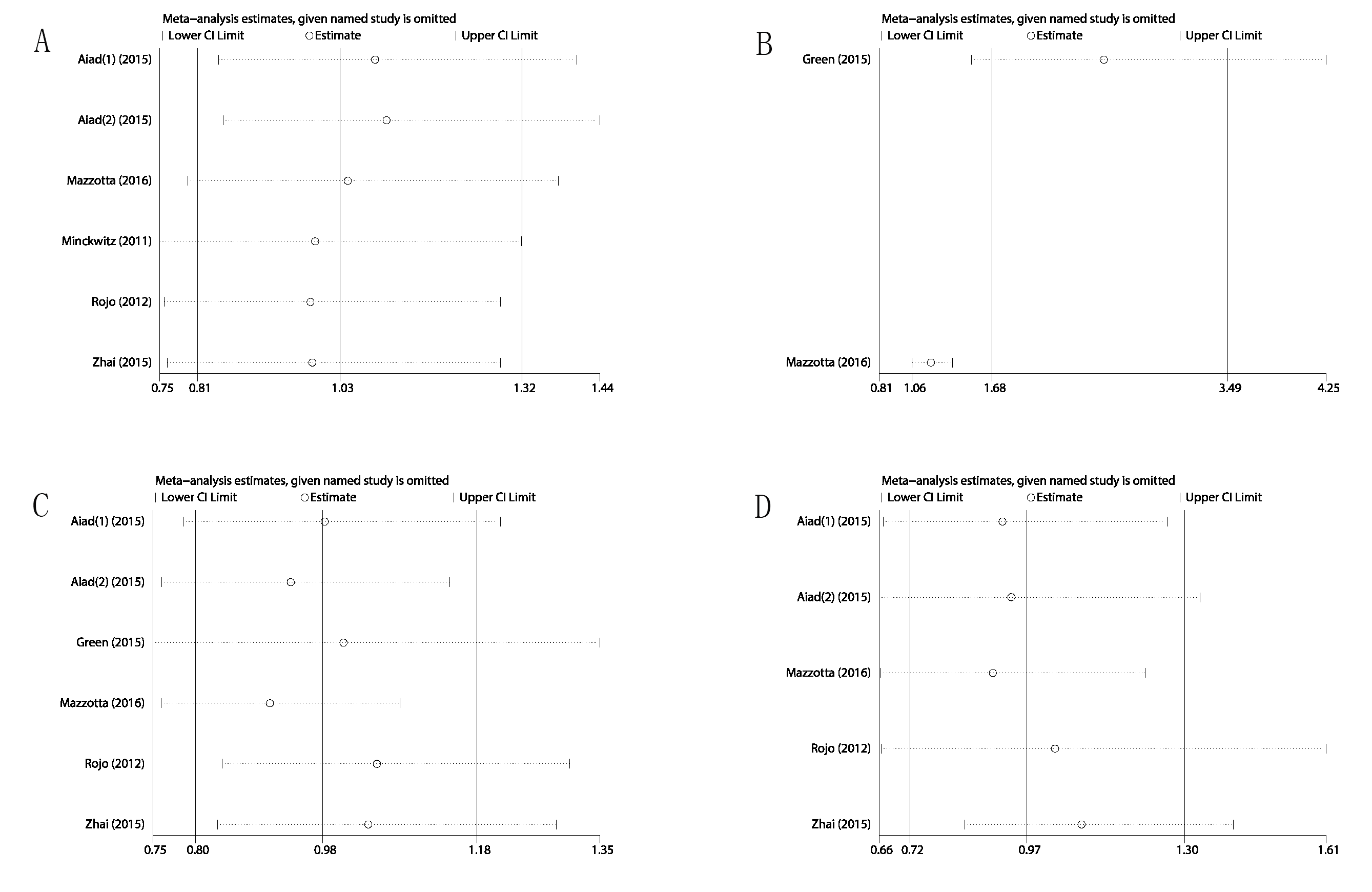

Supplement: S3 Fig — (TIF) [file pone.0172413.s003.tif]

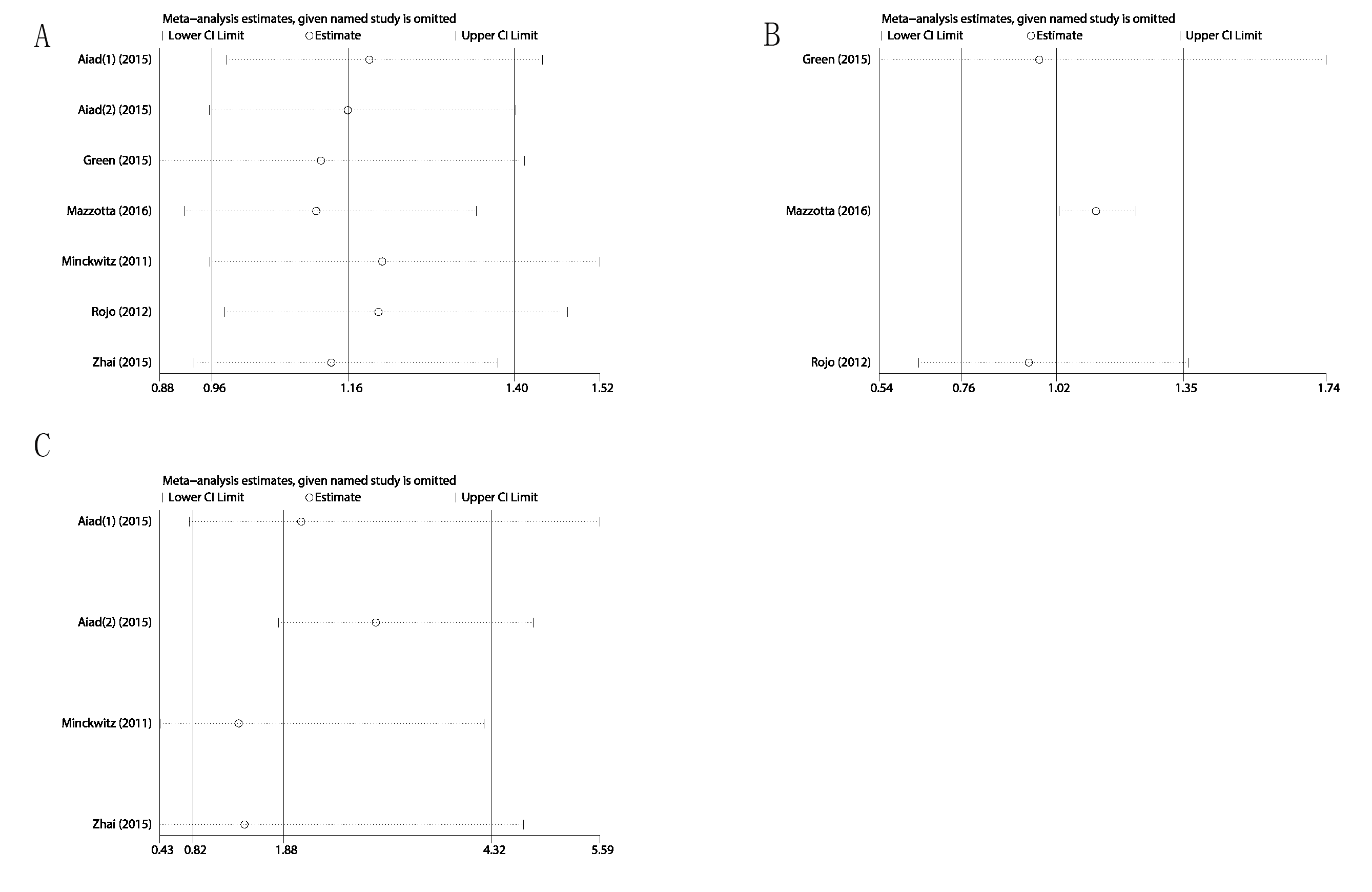

Supplement: S4 Fig — (TIF) [file pone.0172413.s004.tif]
