# Supplementary material for: Prognostic and clinicopathological value of poly (adenosine diphosphate-ribose) polymerase expression in breast cancer: A meta-analysis
Source: PLoS One. 2017 Feb 17;12(2):e0172413. doi: 10.1371/journal.pone.0172413 (PMC5315304; doi:10.1371/journal.pone.0172413)
Supplement: S2 File — (DOC) [file pone.0172413.s006.doc]

**Pubmed:**

Search (((Search ("Breast Neoplasms"[Mesh]) OR (((((((((((((((((((((((((((Breast Neoplasm) OR Neoplasm, Breast) OR Neoplasms, Breast) OR Tumors, Breast) OR Breast Tumors) OR Breast Tumor) OR Tumor, Breast) OR Mammary Neoplasms, Human) OR Human Mammary Neoplasm) OR Human Mammary Neoplasms) OR Neoplasm, Human Mammary) OR Neoplasms, Human Mammary) OR Mammary Neoplasm, Human) OR Mammary Carcinoma, Human) OR Carcinoma, Human Mammary) OR Carcinomas, Human Mammary) OR Human Mammary Carcinomas) OR Mammary Carcinomas, Human) OR Human Mammary Carcinoma) OR Breast Cancer) OR Cancer, Breast) OR Cancer of Breast) OR Mammary Cancer) OR Malignant Neoplasm of Breast) OR Malignant Tumor of Breast) OR Breast Carcinoma) OR Cancer of the Breast))) AND PARP) AND (Search (((pathological complete response) OR pCR)) OR ((((overall survival) OR OS) OR disease-free survival) OR DFS)) (368)

**Embase:**

#1 'breast cancer'/exp 353,930

#2 'breast neoplasms'/exp 423,748

#3 'breast carcinoma'/exp 59,872

#4 #1 OR #2 OR #3 423,748

#5 'parp' 16,969

#6 'pathological complete response' 2,607

#7 'pcr' 548,513

#8 'overall survival'/exp 165,118

#9 'os' 106,982

#10 'disease-free survival'/exp 50,768

#11 'dfs' 18,148

#12 #6 OR #7 OR #8 OR #9 OR #10 OR #11 785,044

#13 #4 AND #5 AND #12 313

**The Cochrane Library:**

.#1MeSH descriptor: [Breast Neoplasms] explode all trees 9702

.#2Breast Neoplasm:ti,ab,kw (Word variations have been searched) 11525

.#3Neoplasms, Breast:ti,ab,kw (Word variations have been searched) 11525

.#4Breast Tumors:ti,ab,kw (Word variations have been searched) 4757

.#5Breast Tumor:ti,ab,kw (Word variations have been searched) 4757

.#6Human Mammary Neoplasm:ti,ab,kw (Word variations have been searched) 275

.#7Breast Cancer:ti,ab,kw (Word variations have been searched) 20388

.#8Cancer, Breast:ti,ab,kw (Word variations have been searched) 20388

.#9Malignant Tumor of Breast:ti,ab,kw (Word variations have been searched) 235

.#10Breast Carcinoma:ti,ab,kw (Word variations have been searched) 2269

.#11 #1 or #2 or #3 or #4 or #5 or #6 or #7 or #8 or #9 or #10 21999

.#12PARP:ti,ab,kw (Word variations have been searched) 95

.#13pathological complete response:ti,ab,kw (Word variations have been searched) 480

.#14pCR:ti,ab,kw (Word variations have been searched) 4350

.#15overall survival:ti,ab,kw (Word variations have been searched) 18290

.#16OS:ti,ab,kw (Word variations have been searched) 6799

.#17disease-free survival:ti,ab,kw (Word variations have been searched) 8824

.#18DFS:ti,ab,kw (Word variations have been searched) 1514

.#19 #13 or #14 or #15 or #16 #17 or #18 23091

.#20 #11 and #12 and #19 16

**Web of Science:**

# 1 (378,274)

TOPIC: (breast cancer) OR TOPIC: (breast neoplasms) OR TOPIC: (breast carcinoma) OR TOPIC: (breast Tumor)

Indexes=SCI-EXPANDED, SSCI, A&HCI, CPCI-S, ESCI, CCR-EXPANDED, IC Timespan=All years

# 2 (11,979)

TOPIC: (PARP) Indexes=SCI-EXPANDED, SSCI, A&HCI, CPCI-S, ESCI, CCR-EXPANDED, IC Timespan=All years

# 3 (550,817)

TOPIC: (pathological complete response) OR TOPIC: (pCR) OR TOPIC: (overall survival) OR TOPIC: (OS) OR TOPIC: (disease-free survival) OR TOPIC: (DFS)

Indexes=SCI-EXPANDED, SSCI, A&HCI, CPCI-S, ESCI, CCR-EXPANDED, IC Timespan=All years

# 4 (150)

#3 AND #2 AND #1

Indexes=SCI-EXPANDED, SSCI, A&HCI, CPCI-S, ESCI, CCR-EXPANDED, IC Timespan=All years
