# Supplementary material for: Prognostic and clinicopathological value of poly (adenosine diphosphate-ribose) polymerase expression in breast cancer: A meta-analysis
Source: PLoS One. 2017 Feb 17;12(2):e0172413. doi: 10.1371/journal.pone.0172413 (PMC5315304; doi:10.1371/journal.pone.0172413)
Supplement: S1 Table — (DOC) [file pone.0172413.s007.doc]

S1 Table. Results of quality assessment

| First author, year | Selection^1^ | | | | Comparability^2^ | Outcome^3^ | | |  |
| --- | --- | --- | --- | --- | --- | --- | --- | --- | --- |
|  | Representativeness of exposed cohort ★ | Selection of non-exposed cohort  ★ | Ascertainment of exposure ★ | No primary outcome was present at start of study ★ | Comparable  on confounder ★★ | Outcome  Assessment ★ | Adequate  follow-up★ | Loss to follow-up★ | Total Score |
| Aiad, 2015 | ★ | ★ | ★ |  | ★★ | ★ | ★ |  | 7 |
| Donizy, 2014 | ★ | ★ | ★ | ★ | ★ | ★ | ★ | ★ | 8 |
| Goncalves, 2011 | ★ | ★ | ★ |  | ★★ | ★ | ★ | ★ | 8 |
| Green, 2015 | ★ | ★ | ★ | ★ | ★ | ★ | ★ | ★ | 8 |
| Mazzotta, 2016 | ★ | ★ | ★ |  | ★★ | ★ | ★ | ★ | 8 |
| Minckwitz, 2011 | ★ | ★ | ★ |  | ★★ | ★ | ★ | ★ | 8 |
| Rojo, 2012 | ★ | ★ | ★ |  | ★★ | ★ | ★ | ★ | 8 |
| Zhai, 2015 | ★ | ★ | ★ |  | ★★ | ★ | ★ |  | 7 |

1“Selection” part includes representativeness of cases, selection of controls, exposure ascertainment, and no death when investigation begin.

2“Comparability” part includes comparable on confounders.

3“Outcome” part includes outcome assessment, adequate follow-up, and loss to follow-up rate.
