# Supplementary material for: Prognostic and clinicopathological value of poly (adenosine diphosphate-ribose) polymerase expression in breast cancer: A meta-analysis
Source: PLoS One. 2017 Feb 17;12(2):e0172413. doi: 10.1371/journal.pone.0172413 (PMC5315304; doi:10.1371/journal.pone.0172413)
Supplement: S2 Table — (DOC) [file pone.0172413.s008.doc]

S2 Table. Results of meta-regression analysis exploring the source of heterogeneity with DFS.

| Covariates | Multivariable analysis | | |
| --- | --- | --- | --- |
|  | Coefficient | SE | P value |
| PARP phenotype | -0.74 | 1.05 | 0.61 |
| Cut-off of PARP | -0.78 | 0.68 | 0.45 |
