# Supplementary material for: Prognostic and clinicopathological value of poly (adenosine diphosphate-ribose) polymerase expression in breast cancer: A meta-analysis
Source: PLoS One. 2017 Feb 17;12(2):e0172413. doi: 10.1371/journal.pone.0172413 (PMC5315304; doi:10.1371/journal.pone.0172413)
Supplement: S3 Table — (DOC) [file pone.0172413.s009.doc]

S3 Table. Results of meta-regression analysis exploring the source of heterogeneity with OS.

| Covariates | Multivariable analysis | | |
| --- | --- | --- | --- |
|  | Coefficient | SE | P value |
| Cancer stage | 0.29 | 0.87 | 0.76 |
| Detection method | -1.72 | 0.58 | 0.06 |
| PARP phenotype | 0.45 | 0.42 | 0.37 |
| Cut-off of PARP | 1.06 | 0.30 | 0.04 |
